# Different kidney function trajectory patterns before dialysis in elderly patients: clinical implications and outcomes

**DOI:** 10.1080/0886022X.2021.1945464

**Published:** 2021-06-30

**Authors:** Josefina Santos, Pedro Oliveira, Milton Severo, Luísa Lobato, António Cabrita, Isabel Fonseca

**Affiliations:** a Nephrology Department, Centro Hospitalar Universitário do Porto (CHUP), Porto, Portugal; b Unit for Multidisciplinary Research in Biomedicine, Institute of Biomedical Sciences Abel Salazar, University of Porto, Porto, Portugal; c EPI Unit, ISPUP – Institute of Public Health, University of Porto, Porto, Portugal; d Department of Population Studies, Institute of Biomedical Sciences Abel Salazar, University of Porto, Porto, Portugal

**Keywords:** CKD, ESKD, outcomes, renal function trajectory

## Abstract

**Background**. Identifying trajectories of kidney disease progression in chronic kidney disease (CKD) patients may help to deliver better care. We aimed to identify and characterize trajectories of renal function decline in CKD patients and to investigate their association with mortality after dialysis.

**Methods.** This retrospective cohort study included 378 CKD patients who initiated dialysis (aged 65 years and over) between 2009 and 2016. Were considered mixed models using linear quadratic and cubic models to define the trajectories, and we used probabilistic clustering procedures. Patient characteristics and care practices at and before dialysis were examined by multivariable multinomial logistic regression. The association of these trajectories with mortality after dialysis was examined using Cox models.

**Results.** Four distinct groups of eGFR trajectories decline before dialysis were identified: slower decline (18.3%), gradual decline (18.3%), early rapid decline (41.2%), and rapid decline (22.2%). Patients with rapid eGFR decline were more likely to have diabetes, more cognitive impairment, to have been hospitalized before dialysis, and were less likely to have received pre-dialysis care compared to the patients with a slower decline. They had a higher risk of death within the first and fourth year after dialysis initiation, and after being more than 4 years in dialysis.

**Conclusions.** There are different patterns of eGFR trajectories before dialysis initiation in the elderly, that may help to identify those who are more likely to experience an accelerated decline in kidney function, with impact on pre ESKD care and in the mortality risk after dialysis.

## Introduction

Chronic kidney disease (CKD) is rising worldwide and has emerged as a serious public health problem, as shown by the increase in mortality and the growing incidence and prevalence of end-stage kidney disease (ESKD) [1], requiring renal replacement therapy (RRT).

Renal function trajectory defined by the change in a patient’s estimated glomerular filtration rate (eGFR) over time is an approach supported by the dynamic changes in kidney function over time, that predicts CKD progression [2,3], and is associated with mortality [4–7].

Although renal function progressively decreases over time, many CKD patients have a prolonged period of non-progression or a non-linear GFR trajectory due to several factors, such as acute kidney injury (AKI) episodes [2], particularly frequent in older patients [7]. Moreover, mortality outweighs the risk of progression to ESKD in the elderly [8], which further complicates the study of patterns of kidney disease progression in this group of patients.

Stratifying CKD patients into different groups according to patterns of renal function trajectory may help to anticipate the optimal timing of nephrology referral, to guide the care of CKD patients.

Since the pioneering work of Mitch et al. [9], kidney function trajectories have been the subject of different research approaches [4,10–13]. Only a few studies have addressed this issue in the elderly [14–16] and in patients who initiate dialysis [4,12,17–19].

Therefore, the aims of this study were to identify and characterize distinct trajectories of renal function decline in CKD patients older than 65 years at dialysis initiation and to investigate the association of these trajectories with mortality after starting dialysis.

## Methods

A retrospective cohort study was conducted in patients, referred to Nephrology Department in Centro Hospitalar Universitario do Porto (CHUP) who started dialysis as their first RRT between January 2009 and December 2016. This hospital is one of the largest tertiary healthcare centers in Northern Portugal, which serves a population of 500 000 inhabitants.

The inclusion criteria for this study were age over 65 years at dialysis initiation and having at least five consecutive serum creatinine measurements, from the first available value at an outpatient visit until dialysis initiation. Patients, who initiated maintenance dialysis due to AKI without CKD, were excluded.

Clinical data included sex, age, weight, height, body mass index (BMI), and associated comorbid conditions, such as diabetes, dyslipidemia, hypertension, smoking status, malignancy, coronary artery disease, congestive heart failure, arrhythmia, peripheral artery disease, and stroke. Laboratory data were collected over time and included: serum creatinine, and urinary protein-to-creatinine ratio. All measurements were performed in the same central laboratory of CHUP using standard biochemical methods.

In the 12 months prior to dialysis initiation, the number of all-cause hospitalizations were collected, including hospitalizations with an inpatient diagnostic code for AKI (ICD-9 codes 584.5–584.9).

Glomerular filtration rate was estimated (eGFR) using the Chronic Kidney Disease Epidemiology (CKD-EPI) 2009 creatinine equation [20]. The etiological diagnosis of CKD was based on the patient’s history, kidney ultrasound, and kidney biopsy, when available.

Cognitive status was evaluated using the Mini-Mental State Examination (MMSE) [21], classified as cognitive impairment if the score was 23 or lower.

Functional dependency was defined as the requiring of assistance for transfer and classified as totally dependent, need assistance for transfer, or autonomous.

A modified version of the Charlson Comorbidity Index (mCCI) [22], that is, by excluding the subject’s age and presence of kidney disease, was calculated.

Variables related to renal care included timing of nephrologist care, vascular access placement (graft/fistula/peritoneal catheter vs. hemodialysis catheter), and whether dialysis was initiated in the hospital.

We also analyze whether there was a diagnostic of AKI associated with inpatient dialysis initiation; AKI was identified by using the criteria of the KDIGO-AKI Work Group guidelines [23].

Vital status was checked for all patients until 31 October 2019.

This study was approved by the Institutional Review Board of Centro Hospitalar do Porto (n°178-DEFI/160-CES), which conducted a scientific and ethical evaluation, and were performed in given the retrospective nature of the study, the Ethics Committee waived the request for informed consent accordance with the national rules and regulations as well as international guidelines. Data were de-identified by removing the patient names and hospital numbers.

### Statistical analysis

To define the trajectories, mixed models using linear quadratic and cubic models were considered. Ciampi et al. [24] refer to the use of clustering to study disease trajectories specifically in the study of longitudinal data where the number of observations or the time between observations may differ across patients. This approach characterizes trajectories in repeated measurements with the assumption that several underlying subpopulations can be detected. It does not require the same number of measurements per patient or the same time points of measurement. We considered for each patient all the eGFR measurements until dialysis initiation.

Probabilistic clustering procedures [25–27] were used to assign the individual trajectories to the different clusters, through the package mclust available as a contributed package from the Comprehensive R Archive Network (CRAN). The number of cluster trajectories was defined based on the Bayesian Information Criterion (BIC).

The percentage of eGFR variation was defined as eGFR at the beginning of the period of follow-up minus eGFR at the end of the period of observation, divided by the value at the beginning.

Demographic and clinical characteristics were summarized as mean and standard deviation (or as a median and interquartile range) for continuous variables and as counts and percentages for categorical variables in trajectory groups. For quantitative variables, one-way ANOVA or the Kruskal–Wallis test was used to compare the different trajectory groups; multiple comparisons were adjusted by the Bonferroni correction.

Univariate logistic regression analysis was used to study the association of each trajectory group with binary patient characteristics and care practices at and before dialysis association. Therefore, one multinomial logistic equation model was used to predict an individual’s probability of belonging to a particular trajectory group based on variables significantly associated with the univariate logistic regression framework. Thus, age, gender, diabetes, proteinuria at baseline, cognitive impairment, and hospitalizations were included in the model to examine if they may predict the group allocation.

Median survival was estimated for each trajectory group using Kaplan–Meier analysis. Multivariable Cox proportional hazard was used to evaluate the association of eGFR trajectory with survival after dialysis initiation, adjusting for patient demographic and clinically relevant factors with trajectories of eGFR decline. The assumption of proportional hazards was checked graphically using the log cumulative hazard plots for death according to subgroups of trajectory eGFR decline. Hazard ratios are presented for three time periods (<1 year, 1–4 years, and >4 years) to fulfill the proportional hazards assumption for the principal predictor variable (trajectory subgroups).

All statistical analyses were performed using R (R Development Core Team 2006) package mclust [25–27] available as a contributed package from the Comprehensive R Archive Network (CRAN) at http://CRAN.R-project.org/ and SPSS 25.0. at an *a priori* significance level of 0.05.

## Results

### Identification of eGFR trajectories decline before dialysis initiation

**Figure 1.**
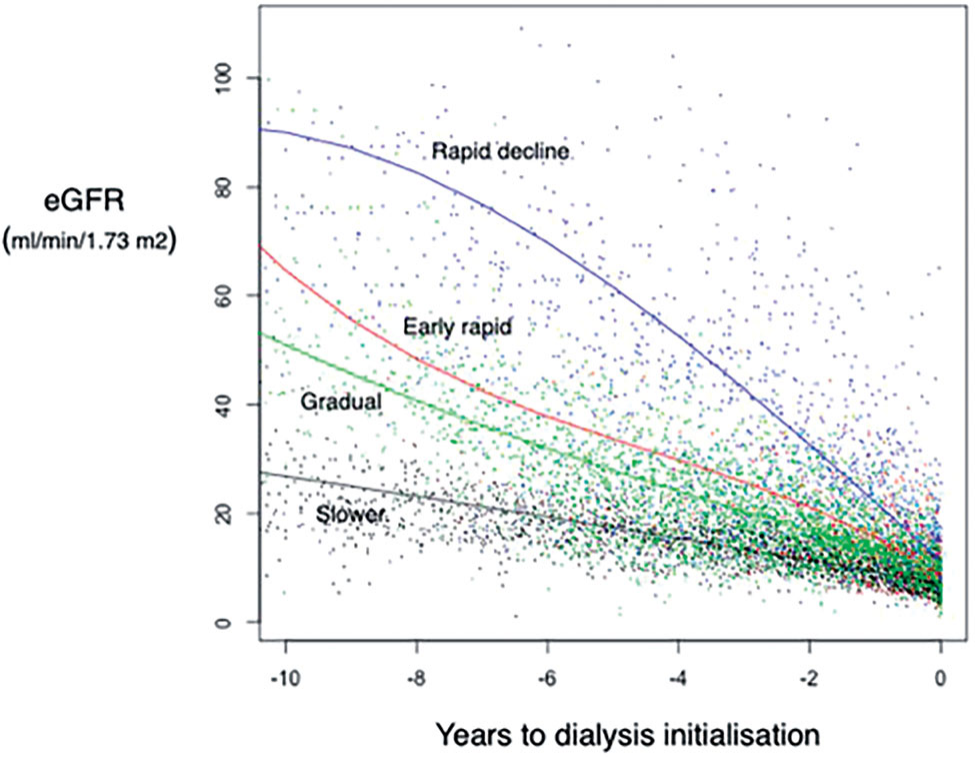
Trajectories decline for the identified groups.

Four distinct groups of eGFR trajectories decline before dialysis initiation were identified: slower eGFR decline (group 1, *n* = 69; 18.3%), gradual eGFR decline (group 2, *n* = 69; 18.3%), early rapid eGFR decline (group 3, *n* = 156; 41.2%) and rapid eGFR decline (group 4, *n* = 84; 22.2%) (Figure 1). Table 1 describes the characteristics of kidney function for each of the identified trajectories.

**Table 1.** Characteristics of each and overall trajectory decline group.

	Overall *n* = 378	Slower eGFR decline (Group 1) *n* = 69	Gradual eGFR decline (Group 2) *n* = 69	Early rapid eGFR decline (Group 3) *n* = 156	Rapid eGFR decline (Group 3) *n* = 84	*p*-Value
SCr measures (*n*)	8252	2074	745	3744	1689	<0.001
Median and IQR	19.0 [11.0–28.0]	26.0 [19.5–38.5]	9.0 [17.0–13.0]	22.0 [14.0–22.8]	16.0 [9.3–29.0]	2-4
						2-3
						2-1
						4-1
						3-1
Follow-up (years)						
Median and IQR	6.0 [3.7–8.6]	9.4 [6.3–11.1]	2.0 [1.4–2.8]	6.3 [4.6–8.5]	6.5 [4.1–7.9]	<0.001
						2-4
						2-3
						2-1
						4-1
						3-1
Time between SCr Measures (years)						
Median and IQR	0.29 [0.22–0.45)	0.30 [0.24–0.43]	0.22 [0.16–0.37]	0.29 [0.23–0.45]	0.31 [0.23–0.60]	<0.001
						2-1
						
						2-3
						2-4
eGFR initial						
(mL/min/1.73 m^2^) Median and IQR	31.1 [20.5–47.7]	22.3 [17.7–29.7]	21.1 [16.8–26.8]	34.0 [23.4–44.0]	61.9 [46.2–76.1]	<0.001
						1-3
						1-4
						2-3
						2-4
						3-4
eGFR final						
(mL/min/1.73 m^2^) Median and IQR	6.6 [5.0–8.5]	5.8 [4.2–7.3]	6.7 [4.9–8.5]	6.7 [5.0–8.6]	7.4 [5.4–9.7]	=0.04
						1-4
						
% eGFR var^a^						
Median and IQR	77.9 [66.9–87.2]	74.1 [64.9–82.2]	69.3 [56.1–77.9]	78.8 [71.1–86.4]	88.3 [78.0–92.2]	<0.001
						2-3
						2-4
						1-4
						3-4

Data expressed as medians and interquartile ranges (IQR).

SCr, serum creatinine; eGFR, estimated glomerular filtration rate eGFR, using the Chronic Kidney Disease Epidemiology.

^a^Defined as eGFR at the beginning of the period of follow-up minus eGFR at the end of the period of observation, divided by the eGFR value at beginning.

The trajectory group with the slower decline eGFR (group 1) was considered as the reference group.

The overall cohort had a median follow-up of 6.0 (3.7–8.6) years, between the first creatinine measurement until dialysis initiation (Table 1).

During the period before dialysis initiation, among the overall cohort, there was a median (IQR) of 19.0 (11.0, 28.0) serum creatinine measurements per patient, and a median eGFR variation (IQR) of 77.9 (66.9, 87.2) percent over a median (IQR) period of 6.0 (3.7, 8.6) years. The median (IQR) eGFR variation in the slower, gradual, early rapid, and rapid decline trajectories groups were 74.1 (64.9, 82.2), 69.3 (56.1, 77.9), 78.8 (71.1, 86.4), and 88.3 (78.0, 92.2) percent, respectively.

### Patient characteristics and nephrology care practices determinants of eGFR trajectories

Table 2 shows baseline characteristics of the overall cohort and according to trajectory group. Table 3 summarizes the nephrology care practices of the overall cohort and stratified by each trajectory’s groups. The patient characteristics and care practices significantly associated with each trajectory are shown in Table 4. The trajectory group with the slower decline eGFR (group 1) was considered as the reference to which other groups were compared.

**Table 2.** Patient characteristics by eGFR trajectory group.

	Overall *n* = 378	Slower eGFR decline (Group 1) *n* = 69	Gradual eGFR decline (Group 2) *n* = 69	Early rapid eGFR decline (Group 3) *n* = 156	Rapid eGFR decline (Group 4) *n* = 84
Age (years), mean; SD	75.4 ± 6.2	76.0 ± 6.2	76.1 ± 6.1	75.3 ± 6.4	74.4 ± 5.9
Age ≥75 years, *n* (%)	193 (51.1)	39 (56.5)	39 (56.5)	82 (52.6)	33 (39.3)
Female, *n* (%)	173 (45.8)	24 (34.8)	33 (47.8)	70 (44.9)	46 (54.8)
Primary renal disease, *n* (%)					
Diabetic nephropathy	142 (37.6)	17 (24.6)	25 (36.2)	63 (40.4)	37 (44.0)
Ischemic nephropathy	61 (16.1)	11 (16.0)	16 (23.2)	25 (16.0)	9 (10.7)
Glomerulonephritis	41 (10.8)	9 (13.0)	5 (7.2)	15 (9.6)	12 (14.3)
ADPKD	21 (5.6)	6 (8.7)	4 (5.8)	9 (5.8)	2 (2.4)
Other	67 (17.7)	11 (16.0)	11 (16.0)	26 (16.7)	19 (22.6)
Unknown etiology	46 (12.2)	15 (21.7)	8 (11.6)	18 (11.5)	5 (6.0)
BMI (kg/m^2^), median and IQR	25.8 [23.5–28.7]	25.3 [24.0–28.3]	26.2 [24.0–28.3]	25.7 [23.5–29.4]	25.8 [22.4–28.2]
<25, *n* (%)	154 (40.7)	30 (43.5)	24 (34.8)	62 (39.7)	34 (40.5)
25–29.9	135 (35.7))	26 (37.7)	30 (43.5)	51(32.7)	31 (36.9)
≥30	68 (18.0)	11 (15.9)	11 (15.9)	33 (21.2)	13 (15.5)
Cognitive impairment, *n* (%)	58 (15.3)	7 (10.1)	9 (13.0)	21 (13.5)	21 (25.0)
Totally dependent for transfer, n,(%)	34 (9.0)	4 (5.8)	5 (7.2)	9 (5.8)	16 (19.0)
Need assistance for transfer, *n* (%)	167 (44.2)	29 (42.0)	32 (46.4)	63 (40.4)	43 (51.2)
Autonomous, *n* (%)	177 (46.8)	36 (52.2)	32 (46.4)	84 (53.8)	25 (29.8)
Institutionalization, *n* (%)	19 (5.0)	3 (4.3)	4 (5.8)	7(4.5)	5 (6.0)
mCCI, median and IQR	4 [2–5]	5 [3–6]	3 [2–5]	4 [2–5]	4 [3–6]
0–2, *n* (%)	112 (29.6)	16 (23.2)	27(39.1)	56 (35.9)	13 (15.5)
3–4	112 (29.6)	16 (23.2)	20 (29.0)	42 (26.9)	34 (40.5)
≥ 5	154 (40.7)	37 (53.6)	22 (31.9)	58 (37.2)	37 (44.0)
Current/former smoking, *n* (%)	86 (22.8)	12 (17.4)	19 (27.5)	40 (25.7)	15 (17.9)
Diabetes, *n* (%)	194 (51.3)	26 (37.7)	35 (50.7)	82 (52.6)	51 (60.7)
Hypertension, *n* (%)	367 (88.6)	69 (100.0)	67 (97.1)	152 (97.4)	66 (78.6)
Dyslipidemia, *n* (%)	335 (89.1)	65 (94.2)	58 (84.0)	146 (93.6)	66 (78.6)
Congestive heart failure, *n* (%)	239 (63.2)	40 (58.0)	44 (63.8)	94 (60.3)	61 (72.6)
Coronary artery disease, *n* (%)	114 (30.2)	15 (21.7)	17(24.6)	51 (32.7)	31 (36.9)
Cardiac arrhythmia, *n* (%)	90 (23.8)	16 (23.2)	8 (11.6)	45 (28.8)	21 (25.0)
Stoke, *n* (%)	117 (31.0)	21 (30.4)	20 (29.0)	50 (32.1)	26 (31.0)
Peripheral vascular disease, *n* (%)	149 (39.4)	27 (39.1)	22 (31.9)	69 (44.2)	30 (35.7)
Cancer, *n* (%)	59 (15.6)	13 (18.8)	13 (18.8)	22 (14.1)	11 (13.1)
COPD, *n* (%)	70 (18.5)	11 (15.9)	17 (24.6)	28 (17.9)	14 (16.7)
Chronic liver disease, *n* (%)	30 (7.9)	4 (5.8)	4 (5.8)	9 (5.8)	13 (15.5)
Autoimmune disease, *n* (%)	15 (4.0)	3 (4.3)	4 (5.8)	5 (3.2)	3 (3.6)
Peptic ulcer, *n* (%)	57 (15.1)	11 (15.9)	9 (13.0)	21 (13.5)	16 (19.0)
Albumin (g/dL), median and IQR	3.7 [3.2–4.1]	3.8 [3.5–4.1]	3.8 [3.5–4.1]	3.7 [3.3–4.0]	3.4 [3.0 − 3.8]
≥3.5, *n* (%)	234 (61.9)	39 (56.5)	54 (78.3)	104 (66.7)	37 (44.0)
3.0–3.49	82 (21.7)	14 (20.3)	9 (13.0)	32 (20.5)	27 (32.2)
<3.0	62 (16.4)	16 (23.2)	6 (8.7)	20 (12.8)	20 (23.8)
uPCr (g/g) at baseline, median and IQR	1.0 [0.3–1.4]	0.8 [0.3–1.6]	1.4 [0.4–3.0]	0.9 [0.2–2.0]	1.7 [0.3 − 3.4]
uPCr ≥ 3.5 (g/g), *n* (%)	48 (13.9)	2 (2.9)	8 (11.6)	21 (13.5)	17 (20.2)
uPCr (g/g), median and IQR^a^	1.8 [0.5–3.7]	1.5 [0.5–2.8]	1.6 [0.3–3.1]	1.7 [0.5–3.7]	2.5 [0.4–6.0]
uPCr ≥ 3.5 (g/g), *n* (%)	102 (27.0)	11 (15.9)	15 (21.7)	43 (27.6)	33 (39.3)
No. of hospitalizations	285 (75.4)	43 (62.3)	52 (75.4)	122 (78.2)	68 (81.0)
Inpatient diagnosis of AKI^b^	258 (68.3)	41 (59.4)	44 (63.8)	112 (71.8)	61 (72.6)

Data expressed as medians and interquartile ranges (IQR) or *n* (%) when appropriate.

ADPKD, Autosomal dominant polycystic kidney disease; BMI, body mass index; mCCI, modified Charlson Comorbidity Index; COPD, chronic obstructive pulmonary disease; uPCr urinary protein-to-creatinine ratio (g/g); AKI, Acute Kidney Injury.

uPCr urinary protein-to-creatinine ratio (g/g) at baseline.

^a^uPCr most recent value before dialysis initiation. ^b^Among patients hospitalized at least once before dialysis initiation.

**Table 3.** Nephrology care practices by eGFR trajectory group.

	Overall *n* = 378	Slower eGFR decline (Group 1) *n* = 69	Gradual eGFR decline (Group 2) *n* = 69	Early rapid eGFR decline (Group 3) *n* = 156	Rapid eGFR decline (Group 3) *n* = 84
Outpatient visit to a nephrologist, *n* (%)	346 (91.5)	69 (100.0)	59 (85.5)	151 (96.8)	67 (79.8)
Time from first nephrology visit to dialysis initiation (months)^a^	47.4 [21.0–92.6]	114.5 [55.2–144.1]	17.8 [9.6–28.7]	54.1 [29.5–89.0]	34.8 [10.3–73.6]
eGFR^b^ ≥ 15 mL/min/1.73 m^2^, *n* (%)	11 (2.9)	0 (0)	1 (1.4)	2 (1.3)	8 (9.5)
Dialysis modality: hemodialysis, *n* (%)	268 (96.4)	62 (89.9)	69 (100.0)	153 (98.0)	84 (100.0)
Access at first dialysis:fistula/graft or PD catheter, *n* (%)	234 (61.9)	52 (75.4)	40 (58.0)	107 (68.6)	35 (41.7)
Impatient dialysis initiation, *n* (%)	213 (56.6)	24 (34.8)	43 (62.3)	85 (54.5)	62 (73.8)
AKI at dialysis initiation^c^, *n* (%)	134 (62.9)	17 (73.9)	27 (62.8)	54 (63.5)	36 (58.1)

Data expressed as medians and interquartile ranges (IQR) or *n* (%) when appropriate.

eGFR, estimated Glomerular Filtration Rate using the Chronic Kidney Disease Epidemiology; PD, peritoneal catheter; AKI, Acute Kidney injury.

^a^Among patients referred to nephrologist; ^b^at dialysis initiation; ^c^Among patients admitted to the hospital at dialysis initiation (*n* = 213).

**Table 4.** Association of patient characteristics and care practices with the eGFR trajectory group.

	Gradual eGFR decline (*n* = 69)	Early rapid eGFR decline (*n* = 156)	Rapid eGFR decline (*n* = 84)	*p* for trend
**Patient Characteristics**				
Age ≥ 75 years	1.00 (0.51–1.96)	0.85 (0.48–1.51)	0.50 (0.26–0.95)*	0.096
Male	0.58 (0.29 − 1.15)	0.66 (0.36–1.18)	0.44 (0.23–0.85)*	0.104
Diabetes	1.70 (0.86–3.35)	1.83 (1.03–3.27)*	2.56 (1.33–4.92)*	0.045*
Coronary artery disease	1.18 (0.53–2.60)	1.75 (0.90–3.39)	2.11 (1.02–4.34)*	0.137
Congestive heart failure	1.28 (0.64–2.53)	1.10 (0.62–1.96)	1.92 (0.98–3.79)	0.208
Cardiac arrhythmia	0.43 (0.17–1.10)	1.34 (0.70–2.59)	1.10 (0.52–2.33)	0.058
Stroke	0.93 (0.45–1.94)	1.08 (0.58–1.99)	1.03 (0.51–2.04)	0.974
Peripheral vascular disease	0.73 (0.36–1.47)	1.23 (0.69–2.20)	0.86 (0.45–1.67)	0.306
Pulmonary disease	1.72 (0.74–4.02)	1.15 (0.54–2.48)	0.90 (0.45 2.50)	0.529
Cancer	1.00 (0.43–2.35)	0.71 (0.33–1.50)	0.65 (0.27–1.56)	0.625
Cognitive impairment	1.33 (0.47–3.80)	1.38 (0.56–3.41)	2.95 (1.17–7.44)*	0.049*
Diabetes as a cause of ESRD *vs*. others	1.74 (0.83–3.63)	2.07 (1.10–3.91)*	2.41 (1.20–4.83)*	0.078
uPCr ≥ 3.5 (g/g)	4.9 (1.00–24.2)*	5.2 (1.19–22.9)*	10.5 (2.31–47.4)*	0.013*
Hospitalized within 1-year before dialysis	1.85 (0.89–3.85)	2.17 (1.17–4.02)*	2.57 (1.24–5.33)*	0.043*
Inpatient diagnosis of AKI^a^	0.41 (0.10–1.66)	0.84 (0.22–3.21)	0.65 (0.16–2.68)	0.473
**Care practices at or before initiation**				
Outpatient visit to a nephrologist^b^	0.65 (0.17–2.40)	0.50 (0.16–1.54)	0.51 (0.1–1.74)	0.653
Vascular access placement^c^	0.52 (0.25–1.09)	0.80 (0.42–1.54)	0.27 (0.13–0.55)*	<0.001*
Inpatient dialysis initiation	3.31 (1.65–6.65)*	2.39 (1.33–4.33)*	5.64 (2.81–11.3)*	<0.001*
AKI at dialysis initiation^d^	0.59 (0.19–1.82)	0.62 (0.22–1.72)	0.49 (0.17–1.41)	0.615

All dependent variables were categorized in yes vs. no. Values shown are odds ratio (95% confidence interval); trajectory reference group is persistently low eGFR (Group 1, *n* = 69). Predictors starred* are those that were statistically significant.

AKI, acute kidney injury; ESRD, end-stage renal disease; uPCr urinary protein-to-creatinine ratio (g/g).

^a^Inpatient diagnosis of AKI among patients hospitalized at least once before starting dialysis initiation. ^b^Outpatient visit to a nephrologist before dialysis initiation. ^c^Vascular access placement among patients who initiated hemodialysis. ^d^Among patients who initiated dialysis during an inpatient admission.

Congestive heart failure, coronary artery disease, arrhythmia, stroke, peripheral vascular disease, pulmonary disease, cancer, an outpatient visit to a nephrologist, inpatient diagnosis of AKI and AKI at dialysis initiation were not associated with being placed into any trajectory compared with the slower eGFR decline group.

Patients with diabetes or diabetes being the cause of ESKD and with hospitalizations within 1 year before dialysis were more likely to be in early rapid (group 3) and rapid decline (group 4) trajectories. Moreover, patients with cognitive impairment and without vascular access were more likely to be in the rapid decline trajectory (group 4). Patients in rapid decline trajectory (group 4) were also younger and included more female patients (Table 4).

Multinomial logistic regression was then conducted to identify the predictors associated with falling into a particular trajectory group, with the distinct trajectory groups used as the dependent variable (Table 5). As in the previous analysis, group 1 trajectory was specified as the reference category.

**Table 5.** Multivariable adjusted multinomial logistic regression analysis for the associations of demographic and clinical relevant factors with trajectories eGFR decline.

Variables	Gradual eGFR decline (Group 2) *n* = 69			Early rapid eGFR decline (Group 3) *n* = 156			Rapid eGFR decline (Group 4) *n* = 84		
	*β*	aOR (95%CI)	*p*-Value	*β*	aOR (95%CI)	*p*-Value	*β*	aOR (95%CI)	*p*-Value
Age (<75 vs.≥75 years)	0.011	1.011 (0.491–2.082)	0.975	0.234	1.264 (0.691–2.310)	0.447	0.837	2.310 (1.104–4.832)	0.026*
Gender (female vs. male)	0.466	1.593 (0.765–3.316)	0.213	0.480	1.616 (0.873–2.992)	0.127	1.136	3.113 (1.475–6.570)	0.003*
Diabetes (yes vs. no)	0.535	1.707 (0.826–3.530)	0.149	0.461	1.585 (0.863–2.913)	0.138	0.895	2.448 (1.159–5.171)	0.019*
uPCr ≥ 3.5 (g/g) at baseline	1.557	4.743 (0.948–23.741)	0.058	1.621	5.058 (1.130–22.64)	0.034*	2.389	10.91 (2.298–51.75)	0.003*
Cognitive impairment (yes vs. no)	0.439	1.551 (0.531–4.529)	0.422	0.193	1.213 (0.472–3.117)	0.689	1.291	3.636 (1.330–9.942)	0.012*
Hospitalized within 1-year before dialysis (yes vs. no)	0.475	1.608 (0.742–3.482)	0.229	0.745	2.106 (1.104–4.017)	0.024*	0.902	2.466 (1.077–5.645)	0.033*

Values show the risk profile (aOR) for each trajectory group compared to trajectory Group 1 (slower decline). Predictors starred* are those that were statistically significant. aOR, adjusted odds ratio in relation to all the other variables in the table; CI, confidence interval.

Women, younger patients (age <75 vs. ≥75 years), diabetic patients, and patients with cognitive impairment were more likely to be included in the rapid decline trajectory (group 4). Patients with heavy proteinuria and patients hospitalized within 1-year before dialysis were more likely to be placed into any of the rapid eGFR decline trajectories (group 3 and group 4), compared to the patients with slower eGFR decline (Table 5).

### Mortality after dialysis initiation according to eGFR decline trajectories

**Figure 2.**
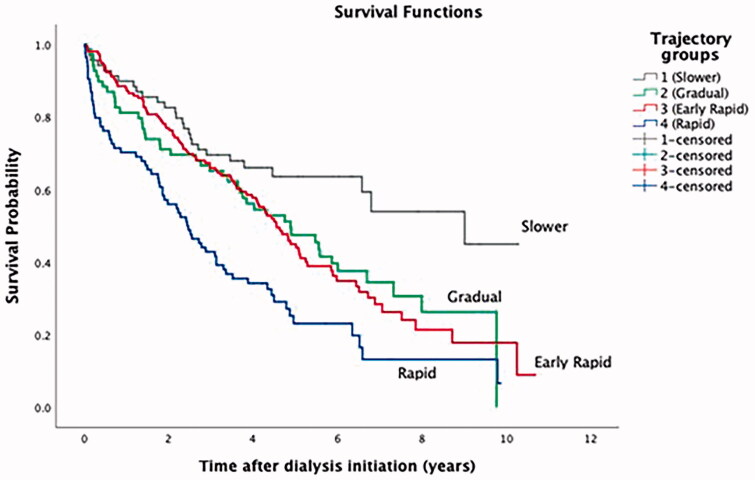
Kaplan–Meier survival curves after dialysis initiation by eGFR trajectory group.

By the end of follow-up in October 2019, 233 patients (62%) had died, with a median survival of 4.4 years (1.8–8.7). Patients with the slower eGFR decline (group 1) had the best median survival (9.0 years) and patients with the more rapid eGFR decline (group 4) had the lowest median survival (2.4 years) (Figure 2).

After adjustment for patient characteristics significant for eGFR trajectories, the trajectory group had no significant impact at the risk for death during the first year after dialysis, compared with the slower decline trajectory (Table 6). However, compared to patients with slower eGFR decline, patients with rapid loss of eGFR (groups 3 and 4) were associated with higher mortality within the first and fourth year after dialysis initiation (HR: 1.805; 95%CI 1.005–3.243, and HR: 3.260; 95%CI 1.693–6.277, for early rapid and rapid eGFR decline, respectively).

**Table 6.** Adjusted risk of death over different periods after dialysis initiation by trajectory group using Cox proportional hazards regression model.

Follow-up Time	Gradual eGFR decline (Group 2) *n* = 69		Early rapid eGFR decline (Group 3) *n* = 156		Rapid eGFR decline (Group 4) *n* = 84)	
	aHR (95%CI)	*p*-Value	aHR (95%CI)	*p*-Value	aHR (95%CI)	*p*-Value
<1 year	0.584 (0.213–1.601)	0.296	0.549 (0.211–1.426)	0.218	1.185 (0.473–2.973)	0.717
1–4 years	1.653 (0.830–3.292)	0.153	1.805 (1.005–3.243)	0.048*	3.260 (1.693–6.277)	<0.001*
>4 years	3.628 (1.171–11.24)	0.026*	4.259 (1.468–12.35)	0.008*	6.347 (1.868–21.56)	0.003*

Values shown are adjusted hazard for death (95% confidence interval); referent group is slower eGFR decline (group 1).

Adjusted for demographic characteristics (age and gender), diabetes, cognitive status, and hospitalization during the 1-year period before dialysis initiation.

eGFR, estimated glomerular filtration rate.; aHR, adjusted hazard ratio; CI, confidence interval. Predictors starred* are those that were statistically significant.

After being more than 4 years in dialysis, patients in trajectories 2, 3, and 4 were at increased significant risk of dying compared with the reference group (group 1) (HR: 3.628; 95%CI 1.171–11.24, HR: 4.259; 95%CI 1.468–12.35, and HR: 6.347; 95%CI 1.868–21.56, respectively) (Table 6). Beyond trajectories, age higher than 75 years was associated with higher mortality. The remaining variables (gender, cognitive status, and hospitalizations within 1-year before dialysis) were not associated with increased mortality risk.

## Discussion

In this current study, four distinct patterns of eGFR decline preceding initiation of chronic dialysis were identified. A significant proportion of patient who initiated dialysis therapy within the follow-up period were those with a median baseline eGFR <25 mL/min/1.73 m^2^ (37%) (group 1 and 2) and had relatively slower slopes, other were patients with higher eGFRs, who had early faster rates of decline (41%) (group 3), whereas 22% of patients with eGFRs >60 mL/min/1.73 m^2^ at baseline, had a catastrophic rate of decline (group 4).

This study was able to identify patient characteristics and care practices that could be determinants of those trajectories. Patients with diabetes or diabetes being the cause of ESKD were more likely to be in eGFR rapid decline trajectories. In a cohort of 18 874 US veterans, Sumida et al. [12], examined the association of eGFR trajectories in late-stage CKD with mortality after dialysis found that patients with fast eGFR decline had a higher prevalence of diabetes mellitus. These results suggest that despite the role of proteinuria in the progression of CKD, chronic hyperglycemia *per si* plays a crucial role in accelerating GFR decline in diabetic patients [28,29], which reinforces the need for tighter monitoring in diabetic patients, whether they have proteinuria or not.

In addition, patients who experienced rapid eGFR decline had higher proteinuria, which is not surprising, considering proteinuria as a highly expected risk factor for the progression of CKD [2,3,30].

O’Hare et al. [4], using data from 5606 Veteran Affairs patients, identified 4 distinct trajectories of eGFR during the 2-year period before dialysis initiation. Like these two previous studies [4,12], we found that patients who experienced more rapid eGFR decline were younger compared with patients who progressed slower. This can be explained by the fact that older patients who survive long enough to reach more advanced stages of CKD are less likely than their younger counterparts to experience fast eGFR decline [4,12].

One particularity of our study is that the rapid eGFR decline group had a higher proportion of women compared with the group that progressed more slowly. Several studies suggest that renal disease progression is faster in men than in women [31]. However, a meta-analysis published in 2003 [32] suggested the progression of renal disease may not be slower in women as compared to men, though most of the women were on post-menopausal age, as in our cohort, considering the lack of protective effect of sex hormones on renal disease progression. In the Chronic Renal Insufficiency Cohort (CRIC) study, despite women having a significantly decreased risk of developing ESKD, after adjusting for demographic and clinical factors there were no significant differences in eGFR slopes between women and men [33]. Thus, it is not clear whether sex is independently associated with faster renal disease progression, or whether the association reflects confounding by imbalances between men and women of non-controlled factors associated with renal disease progression.

We found that patients who experienced rapid eGFR decline had more cognitive impairment than patients with a slower decline. Cognitive impairment is remarkably prevalent in older CKD patients [34] and the celerity of eGFR decline had an increased risk of cognitive deterioration. In addition, by segregating CKD patients with cognitive impairment at baseline we may also be able to discover other important association, as a rapid decline in kidney function exhibit by these patients is thought to be a marker of a greater vascular injury, which supports the hypothesis of a cognition-kidney axis [35].

It has also been shown that cognitive impairment is associated with decreased medical care compliance, decreased adherence to medications, and, hence, could contribute to faster progression to ESKD [36]. Finally, cognitive impairment is a component of frailty, which has been independently associated with an increased risk of decline of kidney function in elderly people [37].

We found that there is an association between cognitive impairment and the rapid decline in function. We are unable to know which one precedes the other. We believe that possibly it will be an interaction of the two clinical situations: CKD and cognitive impairment.

Trajectories of kidney function decline have been identified as independent predictors of ESKD [38,39], so they may hold important implications for the optimal timing of RRT preparation. In our cohort, patients without vascular access and who had inpatient dialysis initiation were more likely to be in the rapid decline trajectory group. These findings are not unpredictable since those with the highest levels of kidney function at beginning of follow-up were least likely to receive pre-dialysis care, maybe to non-recognition of their potential for rapid progress.

Although there is no universally accepted definition for optimal timely evaluation, considered as adequate to allow for patient and family information, preparation for RRT (e.g., vascular access placement), a period of 12 months seems reasonable to provide acceptable nephrology care [40]. In our study, the level of eGFR approximately 12 months before dialysis initiation was 10 mL/min/1.73m^2^ for patients with slower eGFR decline, 15 mL/min/1.73 m^2^ for patients with gradual eGFR decline, around 20 mL/min/1.73 m^2^ for patients with early rapid eGFR decline, and around 25 mL/min/1.73 m^2^ for patients with rapid eGFR decline, respectively (Figure 1). As in other groups [4], these results serve to remind that patients who reach ESKD do so in various ways and suggest the need for more flexible approaches to preparation for RRT with awareness of the heterogeneity of eGFR trajectories.

Our results showed that patients with rapid eGFR decline trajectories were more likely to have been hospitalized within 1-year before starting dialysis. In a cohort of US veterans, with CKD stage 3A, Xie et al. [41], founded that a steeper decline in kidney function was associated with a higher risk of hospitalizations, readmissions, and prolonged length of hospital stay. It is likely that higher rates of hospitalization were attributable in part to deteriorating kidney function and its complications, or that the rate of kidney function decline may be a surrogate marker of poor overall health.

Episodes of AKI in CKD patients are associated with a more rapid transition between stages of CKD and increased risk for progression ESKD [42], particularly in the elderly [43]. In our work, inpatient diagnosis of AKI and the presence of AKI at dialysis initiation were not associated with being placed into any trajectory compared with the slower eGFR decline. This may be related to the fact that AKI episodes were very frequent among all our patients.

Several studies have demonstrated strong associations between the decline in eGFR and the risk of cardiovascular disease and mortality among CKD patients [6,7,38]. However, only a few studies have examined the association of different changes in eGFR with mortality following dialysis initiation [4,12,17].

In our cohort, patients with rapid loss of kidney function had a 3.6-fold increase in adjusted risk for death within the first and fourth year after dialysis initiation. The impact on mortality was maintained after being more than 4 years in dialysis, in whom patients in gradual, early rapid and rapid trajectory decline were at increased significant risk of dying compared with the slower trajectory group (3.6-,4.2- and 6.2-fold higher risk, respectively).

Hsu et al. [17] in the CRIC study, revealed that an abrupt decline in kidney function, defined as having an eGFR > 30 mL/min/1.73 m^2^ at 3 months prior to the start of dialysis, was associated with a 3-fold higher risk for death within the first year after dialysis initiation. O’Hare et al. [8] were able to demonstrate that those patients with the accelerated or abrupt loss of eGFR before dialysis initiation were at nearly twice the risk for dying during the first year after initiation, compared with those patients with persistently low levels of eGFR. Similarly, Sumida et al. [15], examined the association of eGFR trajectories in late-stage CKD with all-cause and cause-specific mortality during the post-ESRD period over a median follow-up of 2.0 years and reported that rapid eGFR decline (less than −5 mL/min/1.73 m^2^/year) was associated with higher all-cause and cardiovascular mortality.

In our cohort, the trajectory group had no significant impact on the risk for death during the first year after dialysis initiation, but the impact of the rapid decline of kidney function in late mortality after dialysis is notorious, and it was even higher after being more than 4 years in dialysis. Although mortality on dialysis therapy has historically been attributed to factors measurable at the time of dialysis itself [44], our results show that rapid loss of eGFR adds information about prognostic over and above known comorbid conditions and confounders.

Another important issue related to the clinical usefulness of the analysis of eGFR trajectory is its usability. The usability reflects the availability of a clinical decision aid, that can be achieved through the availability of electronic health record systems’ use of analytics and data visualization tools in the clinical environment [45].

Our study has several strengths, including detailed population phenotype characterization, using all available measurements of renal function. We provided a description of different chronic trajectories of kidney function leading up to dialysis initiation through the entire spectrum of CKD progression, not only at transitioning period to dialysis. In this study, we assessed kidney function trajectories using a median of 19.0 [11.0–28.0] eGFR assessments over a median duration of 6.0 [3.7–8.6] years, which improves the risk prediction.

There are certain limitations to our research. First, this is a single-center study. We also examine only those patients who ultimately ended up on dialysis therapy, so our results are limited by survivor bias.

## Conclusion

In conclusion, it was observed different patterns of eGFR trajectories before dialysis initiation and identified associated demographic and clinical factors that may help to identify those who are more likely to experience an accelerated decline in kidney function, with an impact on the risk for post-dialysis mortality. The identification and risk stratification of patients at the highest risk for worse outcomes, based on trajectory analysis of eGFR, may help to guide decision support systems and inform the patient in a shared decision-making process.

These findings suggest that the incorporation of the eGFR trajectory into clinical practice will guide shared decision-making on pre EKRD care and prediction of mortality after dialysis initiation.
